# Women football observers’ experiences: a perspective from system justification and glass ceiling

**DOI:** 10.3389/fpsyg.2024.1495843

**Published:** 2024-11-28

**Authors:** Yeliz Eratli Şirin, Günseli ÖZ

**Affiliations:** ^1^Sports Management Department, Faculty of Sports Sciences, Cukurova University, Adana, Türkiye; ^2^Department of Physical Education and Sports, Health Science Institute, Cukurova University, Adana, Türkiye

**Keywords:** football female observers, system justification, glass ceiling, organizational psychology, social pressure

## Abstract

**Introduction:**

The legitimization theory of the system explains that despite people’s inherent drive towards personal and group interests, they tend to support social systems. Understanding the sources of social pressure and the glass ceiling perceptions of female football observers is the main aim of this study in terms of examining attitudes within the legitimized system. To this end, the study sought to answer how female football observers are constructed in a marginalized position within the male-dominated football culture through sources of social pressure, and how they accommodate the acceptance of legitimizing the system despite encountering glass ceiling barriers in the context of Turkey.

**Method:**

Data for the research were collected through in-depth individual interviews (9 female observers) using qualitative research methods. The collected data were analyzed using thematic analysis. After a comprehensive analysis of the interview transcripts, four themes emerged:

**Findings:**

Gender-based positive and negative experiences; Glass Ceiling: Organizational Factors; Legitimization of Hierarchy-Reducing Myths; and Hope for Future Intragroup Progress within the Current System.

**Recommendations:**

Our analysis reveals that female football observers, identified as match officials, indicate changes in authoritarian attitudes within recent institutional policies during their stadium experiences, characterized towards a more rational, female observer, and referee-focused stance. Additionally, entrenched stereotypes and legitimizing myths reducing hierarchy appear to generally establish legitimacy for female match officiating within the legitimization process. The research results suggest the importance for policymakers in the central referee committee in Turkey to consider the dimensions of legitimization both verbally and in writing when creating policies regarding women.

## 1 Introduction

The increase in the popularity of women’s football over the past 20 years, within the historically male-dominated world of football, has been documented in various academic studies (Cleland et al., 2020; Bell, 2019). Against this backdrop, the progress women have made in football in recent years is remarkable, and the participation of girls and women in the game has seen impressive growth (Clarkson et al., 2020). Despite these developments, an examination of women’s presence in football, both globally and in Turkey, reveals that the representation of women in leadership, management, and decision-making positions remains significantly low (Caudwell, 2011; Clarkson et al., 2019). Football has a history of structurally excluding women (Williams, 2003) and plays a major role in producing and reinforcing dominant concepts of masculinity in contemporary popular culture (Cleland et al., 2020).

Women have been marginalized within the football community due to gender-based power dynamics and continue to be underrepresented in roles such as refereeing, observation, and other leadership positions. However, their presence and success can challenge the masculinity of sports environments and the historically gendered nature of sports, inspiring innovative changes in leadership approaches (Nordstrom et al., 2016). Success in sports is often attributed to excellence in both the physical and mental domains of performance (Metin and Eratlı, 2022). By overcoming biases and breaking stereotypes, female referees and observers can contribute to a more inclusive and progressive sports environment. Through these prevailing ideologies and stereotypes, female observers, particularly those from disadvantaged groups, internalize their status during the process of legitimizing the system, perceiving the existing structure as legitimate and fair. System justification theory posits that supportive ideologies and stereotypes are used to maintain the status quo in society. When men who support the system feel threatened by women’s structural empowerment, they may resort to various strategies to reaffirm the legitimacy of the gender hierarchy (Şeker and Direkçi, 2022).

In addition to the obstacles arising from the male-dominated nature of football culture, women in decision-making positions face various challenges due to the glass ceiling and societal pressures (Flint, 2012). The glass ceiling, an invisible barrier that prevents women from advancing beyond a certain level in their careers, is one of the most significant factors limiting the professional development of female football referees. The perception of the glass ceiling among female football referees often revolves around gender discrimination and the struggle to exist in a male-dominated sector (Tingle et al., 2014).

The high number of male referees and their more frequent assignments to top-level matches are factors that hinder the progress of female referees. Regardless of their abilities and achievements, female referees often do not officiate important matches or are assigned to less prestigious leagues. For instance, when examining the number of referees and observers in the league categories of the Turkish Football Federation’s 2023–2024 football season Central Referee Board; out of a total of 1,345 referees, 102 (7.6%) were female, and 1,243 (92.4%) were male. Among the 509 observers, 2.82% were female, and 97.2% were male (TFF, 2024). In the top-tier Super League, while 26 male referees were assigned, no female referees were represented in this category. When examining the role of football observers, it was found that no female observers were present in the Super League, and only one female referee observer was present in the A class. These results indicate that women face the glass ceiling in decision-making positions and are not adequately represented in decision-making mechanisms. Lale Orta, Turkey’s first female football coach and first female football referee, became the first female president of the Turkish Football Federation’s Central Referee Board in January 2023. However, her tenure lasted only 6 months, and she was dismissed in July 2023. Her short tenure serves as evidence of the difficulties women face in maintaining upper management positions in sports. Similarly, in the context of refereeing and observation within the English Premier League, despite a 72% increase in the number of qualified female referees between 2016 and 2020 (Stimpson, 2020), there remains a significant underrepresentation of women, particularly in male football. Rebecca Welch made national headlines in early 2021 as the first female referee to officiate a men’s match in the English Premier League (MacInnes, 2021).

Previous studies on women in football have predominantly focused on the opportunities for girls and women to play the game (Pfister, 2015; Black and Fielding-Lloyd, 2019) and the representation of women in coaching (Burton, 2015; Norman et al., 2018). Few studies have addressed the experiences of women in football refereeing and observation. Jones and Edwards (2013), Forbes et al. (2015), and Reid and Dallaire (2019) have highlighted the conspicuous absence of women in refereeing and observation roles, suggesting that gender inequalities in refereeing may be even more pronounced than those in coaching. It is assumed that women lack the knowledge required to make correct decisions, do not embody the assertive tendencies needed to control the game, and lack the physical abilities to “keep up” with the game, especially in elite men’s football.

Based on the results of these studies, it is evident that while women have undoubtedly made significant progress in terms of participation as players, the same cannot be said for their involvement in decision-making roles (refereeing, observation, etc.) in football. These studies represent a small body of work on female referees and observers, and there are very few studies on female football observers, particularly in Turkey. This research will address the underrepresentation of female football referee observers, aiming to both uncover the reasons for their minority status and develop strategies for addressing the pressures and challenges they face, thus promoting gender equality in football environments. The guiding questions of this research on female football observers are as follows:

1. What are the experiences of female football observers regarding social pressure and system legitimization within the male-dominated football culture?
2. What are the perceptions of the glass ceiling among female referees in the football refereeing community?

In light of these questions, the research aims to explore how female football observers in Turkey adapt to the acceptance of system justification in their experiences of social pressure and the glass ceiling. For this reason, this study focuses on the experiences of women referees and, particularly, observers in football, emphasizing the insidious and persistent nature of system justification, the sources of social pressure inherent in football culture, and their struggles with glass ceiling barriers.

### 1.1 Literature

#### 1.1.1 System justification theory (SJT)

System Justification Theory suggests that individuals are motivated to justify existing social inequalities and legitimize the socio-political system that governs them (Jost et al., 2004). When the system is perceived as stable and legitimate, it is highly supported, and dominant group members adopt hierarchy-enhancing attitudes as long as they see their socio-political system as legitimate, as this helps protect their group-based interests (Laurin et al., 2013). The theory explains why people tend to sustain the existing system, even when it harms them or their group, and how they perceive the system as legitimate and fair (Jost et al., 2014). During the process of system justification, individuals—especially disadvantaged group members—internalize their own status through existing ideologies and stereotypes, perceiving the current structure as legitimate and fair (Jost and ve Hunyady, 2005). Individuals’ evaluations of the system are influenced by their existential, epistemic, and social needs (Jolley et al., 2018).

When individuals perceive threats that could disrupt the structure of the system they live in, they experience negative emotions such as fear and anxiety. A high tendency to justify the system brings about negative attitudes toward change. Moreover, advantaged group members rationalize their privileged positions through a strong tendency to justify the system, while disadvantaged group members tend to blame themselves rather than the system for their circumstances, accepting inequality (Wakslak et al., 2007).

System Justification Theory uses ideologies and stereotypes that support the maintenance of existing systems in society. In this context, women should demonstrate the strongest system defense when the hierarchy is perceived as stable. Furthermore, System Justification Theory proposes that when people face socio-political threats, they engage in psychological processes to restore the legitimacy of the status quo (Lau et al., 2008). Therefore, dominant group members (men) may display heightened system defenses when the gender hierarchy is threatened, as this helps preserve the status quo. Men who defend the system might employ various strategies to re-legitimize the gender hierarchy when they feel threatened by women’s structural empowerment. Potential defense mechanisms include showing anger and resentment towards women (hostile sexism), attributing positive traits to their own group that legitimize existing gender roles (benevolent sexism), and adopting polarized views about traditional and non-traditional women. These views might include negative stereotypes about women fighting for social change (e.g., feminists) and positive stereotypes about stay-at-home mothers (Kuchynka, 2015).

#### 1.1.2 Social pressure in sport

Social Dominance Theory is a framework based on the fundamental observation that all human societies tend to establish group-based social hierarchies. It aims to explain why societies are organized around group-based social hierarchies. The theory focuses on the role of intergroup prejudice, social roles, cultural ideologies, and psychological tendencies in sustaining social hierarchies through discriminatory behaviors (Pratto et al., 2000).

According to Social Dominance Theory, intergroup inequalities are shaped not only by power but also by legitimizing myths that influence the behaviors of institutions, groups, and individuals. Legitimizing myths are a set of shared values, attitudes, beliefs, stereotypes, and cultural ideologies (Sidanius et al., 2000). Social Dominance Theory categorizes legitimizing myths into two types: hierarchy-enhancing legitimizing myths and hierarchy-attenuating legitimizing myths. Hierarchy-enhancing legitimizing myths include all moral and intellectual doctrines that justify group-based oppression and inequalities. Examples of hierarchy-enhancing myths include racism, sexism, stereotypes, the belief in a just world, nationalism, and the belief in karma (Sidanius et al., 2004).

On the other hand, certain ideologies and approaches challenge social dominance. These ideologies are known as hierarchy-attenuating legitimizing myths. Examples of hierarchy-attenuating legitimizing myths include political doctrines such as social democracy, socialism, and communism, religious teachings that advocate for the protection of the poor, and humanistic teachings like feminism and human rights (Pratto et al., 2006). The maintenance of hierarchy-enhancing legitimizing myths is associated with a high social dominance orientation, whereas the maintenance of hierarchy-attenuating legitimizing myths is related to a low social dominance orientation (Pratto et al., 1994).

Based on these explanations, social pressure has always played a critical role in shaping individuals’ behavior across various domains and has influenced the outcome of important events. One of the environments where the phenomenon of social interaction and pressure is most evident is in sports. For instance, the preferences of a specific group, such as the crowd in a football match, can significantly impact the behavior and decisions of the referee or observer. In many cases, individuals are encouraged by social pressure to make decisions that may go against their personal inclinations. These decisions aim to align with the expectations of the group and avoid criticism. Generally, a referee is under more scrutiny than the players on the field, and when the outcome is not accepted by a team and its supporters, the referee figure is questioned and can become a scapegoat. Therefore, referees become targets of social pressure from players, coaches, and especially spectators (Corrado, 2011).

#### 1.1.3 The Glass Wall

The term “Glass Wall” typically refers to an invisible artificial barrier that prevents individuals with the appropriate qualifications from advancing into leadership positions within organizations (Baumgartner and Schneider, 2010). It has been described as a strong and invisible barrier that hinders women’s career progression and their achievement of equality with men (Khalid and Aftab, 2023). These barriers have been categorized into three main areas (Karaca, 2007). These categories include:

- *Barriers arising from individual factors*: These involve women’s multiple roles and personal preferences and perceptions (e.g., women’s roles related to home responsibilities, the challenges of balancing work and family life, role conflicts, internalizing societal values without questioning, the belief that the system cannot be changed, and feeling obligated to support the system).
- *Barriers arising from organizational factors*: Organizational culture and policies, lack of mentors, and exclusion from communication networks form the organizational barriers.
- *Barriers arising from societal factors*: Stereotypes and professional segregation form the societal barriers.

The “Glass Ceiling” refers to all invisible barriers that prevent women from reaching senior management positions due to their gender. Since the inclusion of women in top management implies a power-sharing dynamic between men and women, male-dominated organizational cultures resist women gaining power. Thus, senior management levels are blocked off for women by a transparent glass ceiling (Singh et al., 2023). One of the primary institutions where male-dominated organizational culture prevails in top management and throughout the ranks is sports organizations. Cunningham and Sagas (2008) argued that women in various sports environments often face hostile, adversarial, and discriminatory work conditions. Researchers have suggested that power within sports organizations is gendered and typically operates in favor of men (Sartore and Cunningham, 2007). Status and power are influenced by the behaviors and attitudes of group or society members, and when arranged around a primary social theme, they can lead to the development of stereotypes and often continue traditional social arrangements and structures (Sartore and Cunningham, 2007). Therefore, sports have played a significant role in the development and perpetuation of gender stereotypes and discrimination. Clopton and Sagas (2009) explored various aspects of the glass ceiling, investigating the perceptions of male and female coaches regarding career and promotion opportunities. Their research revealed that even the perception of career and promotion limitations could be a predictor of leaving the profession.

There are very few studies in the literature that examine female coaches and women in leadership roles in sports and provide a critical perspective on the institutional and organizational structures of sports (Norman, 2014). Additionally, only a few studies (Jones and Edwards, 2013; Drury et al., 2022) have highlighted the noticeable absence of women in football refereeing and observer roles, suggesting that gender inequalities in refereeing and observing may be even more pronounced than those in coaching and managerial positions.

## 2 Method

This research was carried out in the phenomenology design, one of the qualitative research designs, using the semi-structured individual interview technique. The phenomenon investigated was “being a female observer in football.” Phenomenology design reveal people’s experiences, attitudes, the meaning of this phenomenon in the individual itself, and the structure and essence of the phenomenon (Creswell, 2007; Merriam, 2009).

### 2.1 Participants

Data for the research were collected through 9 female observers in Turkey. The schools were selected using purposeful sampling method, which is of the purposeful sampling methods.

During the six-month data collection process, a total of 9 certified female observers, aged between 37 and 54, with a minimum of 5 years and a maximum of 13 years of experience (an average of 9.2 years) as observers, were included in the study. Two women had been observers for 5–6 years, four women for 8–10 years, and three women for 11–13 years. Regarding the participants’ marital status, it was found that 4 participants were married and 5 were single. All 9 female observers had served in various leagues in Turkey, including the Amateur League, BAL League, Women’s Super League, 3rd League, and 2nd League (see Table 1).

**Table 1:** The demographic information of the participants is presented in the table below.

Pseudonym	Age	Interview duration	Educational status	Marital status	Number of years officiated	level officiated	Number of years observership	Leagues in which he worked as an observer
Nilay	53	50 min	Bachelor degree	Single	9 years	Class C 1.2.3. League RAL League	12 years	Amateur League RAL League Women’s Super League 3rd League 2nd League
Oya	51	01.15	Bachelor degree	Single	11 years	Regional RAL Women Super League	11 years	Amateur League Youth Teams RAL League 3rd League
Mine	46	01.20	Bachelor degree	Married	12 years	Regional RAL League Women Super League 3rd League	13 years	Amateur League RAL League Women’s Super League 3rd League
Zeynep	38	01.10	Bachelor degree	Married	10 years B	Matches in all provinces Regional RAL League Women Super League	10 years	Amateur League RAL League Women’s Super League
Simge	37	01.15.	Bachelor degree	Single	15 years C	Regional RAL Women Super League	5 years	Amateur League RAL League Women’s Super League
Ecem	54	55 min	Bachelor degree	Married	6 years	Regional RAL Women Super League	8 years	Amateur League RAL League Women’s Super League 3rd League
Sude	43	50 min	Bachelor degree	Married	10 years	Regional RAL	6 years	Amateur League RAL League
Ayşe	48	30 min	Bachelor degree	Single	16 years	FIFA MHK Regional RAL Women Super League	8 years	RAL League Women’s Super League 3rd League
Nükhet	46	01.00.	Master’s degree	Single	8 years	Regional RAL Women Super League	10 years	RAL League Women’s Super League 3rd League Women 1st League 2nd League

### 2.2 Data collection

The research data were collected using a semi-structured interview form. Accordingly, female observers answered open-ended questions, and further inquiry questions were used to encourage detailed answers. The interview questions were created after reviewing the relevant literature on system justıfıcatıon, glass ceılıng and social pressure, and receiving the opinions of an expert. Interviews were held after making appointments with the female observers. All interviews were conducted online on the date determined by the participants (Whatsapp Web). Interviews lasted approximately 45 min. The participants’ identities were kept secret in the study, and code names were used. The interviews were audio-recorded after getting the permission of the participants. Some interview questions are as follow:

- What does it mean to be a female football observer in Turkey? How do you evaluate the meaning of being a female football observer in Turkey in terms of opportunities and challenges?
- Could you talk about the social pressures related to your profession (such as pressure from the crowd, coaches, players, etc.)? What kind of difficulties do these pressures cause while performing your job?
- As a female observer, what obstacles have you faced while climbing the career ladder?
- In your opinion, what is society’s perspective on women and men? (What do you think about gender equality?)
- When comparing female football referees and observers to their male counterparts, what do you think are the advantages and disadvantages in your profession?
- Do you feel that you do not have equal access to rights and opportunities in the football observing profession due to your gender? How?

### 2.3 Data analysis

The collected data were analyzed using thematic analysis. We used the six steps proposed by Braun and Clarke (2006) to conduct an interpretive thematic analysis. First, familiarization with data—the data were organized in the transcripts. Then authors read and re-read transcripts to become familiar with the content. Second, the generation of initial codes—authors and two colleagues from the sports sciences field (one sport management, and one gender studies researcher) separately identified features in a systematic manner using general codes across the data set. Third, searching for themes—the researchers met and discussed their preliminary codes and compared interpretations of further themes. Fourth, reviewing themes—the coded data were developed by consensus into a thematic map, whereby the researchers considered the arrangement of themes and subthemes. Fifth, defining and naming themes—to refine all themes, definitions were derived for each label. Sixth, producing the report—each theme’s title was amended to reflect the study’s data and these were related to the research questions and the literature (Braun and Clarke, 2006). The study received ethical approval from the Ethics Committee of Çukurova University (23/02/2024 and decision number 141).

### 2.4 Credibility

Before the interviews, we made them sign a voluntary participation form and asked them again during the interviews. We explained their rights to end the interview at any time and to skip any question they did not want to answer. We kept the identities of all participants secret and gave them pseudonyms. We shared the transcripts of the research with them and told them that we would remove any parts they did not want us to use in the analysis. We used triangulation researcher (two researchers made independent evaluations during the analysis of data) to ensure the credibility of the study (Denzin, 2012). Expert opinions were obtained in creating the form, and pilot applications were made to ensure the face and content validity of the interview forms. The pilot applications were not included in the study; they were only performed to test whether the questions were easy to understand or not.

## 3 Findings and results

The findings obtained from the study are presented under four themes. These are:

Theme 1: Gender-based positive and negative experiences.
Theme 2: Glass Ceiling: Organizational Factors.
Theme 3: Legitimization of Hierarchy-Reducing Myths.
Theme 4: Hope for Future Intragroup Progress within the Current System.

### 3.1 Theme 1: gender-based positive and negative experiences

The findings under this theme suggest that participants have experienced negative situations due to the perception that football refereeing and observation are considered “men’s work.” They particularly emphasized that the reactions from fans regarding decisions not made in their favor often involved sexist remarks such as “Go home and wash the dishes.” Ecem mentioned: “For example, during matches: ‘A female observer? How could there be a female observer?’…” Ayşe also added, “… female observers are considered adequate for the profession, but are not assigned to prestigious matches like those in the Super League. While we are accepted into these leagues as regional observers, we are not assigned to major matches such as Fenerbahçe games.”

The fact that female observers are not assigned to prestigious matches due to their gender and are subjected to sexist stereotypes has significant implications, both economically and in terms of their status. Gender stereotypes in sports persist in Turkey, as they do globally. This situation leads to women facing prejudices in sports that are coded as “masculine.” For example, studies in the literature reveal that women are perceived as the “other” gender in sports such as basketball (Şahin et al., 2020), boxing (Emir et al., 2015), and combat sports (Kavasoğlu and Yaşar, 2016), and that they experience prejudice, inequality, and discrimination. Reid & Dallaire (2018) also stated that the scarcity of female referees puts them in a paradoxical position: they are viewed as a spectacle and a focal point, but at the same time, they are marginalized and discredited.

The findings of this research also show that football referees and observers are subjected to similar gendered prejudices. From the perspective of system justification theory, these findings remind us that instead of questioning the legitimacy of roles or positions assigned by the system, individuals often define themselves and others based on characteristics that align with these roles or positions, whether positive or negative. Therefore, stereotypes that emerge at this stage serve as a tool for legitimizing the system (Jost and Banaji, 1994). For example, stereotypes such as “incompetent, dirty, unintelligent, untrustworthy” directed at individuals in a certain profession are used to justify their unequal economic share and their lower status in the hierarchy (Jost and Banaji, 1994). These mechanisms also require women athletes to adopt and conform to masculine sports culture and continually prove themselves in order to advance in the hierarchy. Meanwhile, the competence of their male counterparts is typically taken for granted (Walker and Bopp, 2011). McKay (1997) in his study examining gender relations in sports organizations where masculine norms are highly valued, explained that women constantly have to perform ‘gender’ under disadvantaged conditions and deal with gendered structures and norms through strategies of adaptation and acceptance. Consequently, female football referees and observers, like other female athletes, may struggle to question the normalized patriarchal and masculine structures and practices associated with their roles and the sports culture itself. As a result, they may adopt gendered practices that are valued in order to gain acceptance and recognition (Welford, 2011).

Despite the above-mentioned prejudices, participants also believe they have certain opportunities due to being women. These include positive experiences such as being treated more hospitably, not being subjected to violence, and being appreciated for making bold decisions in challenging matches, compared to their male counterparts. They specifically mentioned receiving support from male referee colleagues, who also acted as mentors when needed. For instance, Simge described her positive experiences, which she believes are due to her gender, as follows: “…we are welcomed more politely by field managers, commissioners, facility supervisors, and football representatives compared to men. There is positive discrimination.”

“…We can also observe positive changes in the Turkish Football Federation’s policies towards women, which are reflected in both the fields and management. For example, having a female representative in the Central Referee Committee, and having a female president of the federation in the past, along with a female member in the committee, and female observers in C and A classifications and female FIFA referees, are indicators of this positive change (Sude).”

International sports organizations (such as IOC, FIFA, UEFA) are conducting significant efforts to ensure gender equality in sports (Şirin, 2021). These efforts assign responsibilities to national sports organizations, such as increasing the number of women in various sports positions. The positive experiences mentioned by the participants may stem from the Turkish Football Federation’s efforts to align with the gender equality policies of organizations like UEFA and FIFA.

### 3.2 Theme 2: glass ceiling: organizational factors

In this theme, participants emphasized that they climbed the career ladder more slowly than their male colleagues due to their gender, were assigned fewer matches, and had significantly lower chances of being appointed to higher league matches. They expressed these experiences of being held back as follows:

Oya: “As women, we are like stepchildren; we have never reached the positions we deserved… those who received worse ratings than me (men) always became Super League assistants.”

Sude also noted: “There is no place for female observers in the stands. Governors and other officials come to the stands, but no specific area is arranged for us to watch the matches. Sometimes we end up sitting among the fans. This is an institutional shortcoming, and as a result, we occasionally feel more pressure from the fans. There have been many instances where I just sat wherever I could find a spot to watch the match.”

The participants stressed that they were institutionally marginalized in the profession of football refereeing and observation due to their gender and that they could not rise to the positions they deserved at the pace they were entitled to. Similar to the findings of this study, the literature also highlights the “glass ceiling” (Claringbould and Knoppers, 2012) encountered by women in sports management positions, the neoliberal approach adopted by sports organizations toward gender equality (Hovden, 2015), and the resistance shown by (male) boards in sports organizations to embracing and maintaining gender balance in sports governance (Knoppers et al., 2022), emphasizing the lack of women in these upper echelons.

### 3.3 Theme 3: legitimization of hierarchy-reducing myths

In the findings discussed above, despite participants expressing that they experienced gender-based prejudice and faced institutional “glass ceiling” barriers, this theme suggests that they do not believe there is institutional gender discrimination. For instance, Oya expressed her thoughts on the matter: “I do not think there is discrimination; I think everyone is evaluated equally. There’s no inequality or discrimination.” Furthermore, in these findings, participants highlighted that compared to previous years, there have been improvements in women’s football in Turkey, with women being in better positions in their profession, and that gender-based prejudice in societal perspectives has decreased. Simge commented on this:

“It’s in a much better position than before. So, people no longer have the same level of prejudice as they used to. Now, women can play football, women can commentate on football, and women can referee.”

Hierarchy-enhancing myths support the continuity of hierarchical structures by portraying inequalities in the distribution of social values as normal, moral, and just, thereby promoting the belief that inequality is legitimate. These myths can include any form of sexism, belief in a just world, heterosexism, nationalism, racism, and stereotypes (Pratto et al., 2006). On the other hand, hierarchy-attenuating myths advocate for equality within the social structure and oppose the inequalities in distribution. Examples include democracy, socialism, communism, humanism, human rights, and feminism (Pratto et al., 2006). This theme reflects participants’ observations of the increasing number of female referees and observers, indicating a shift towards greater representation compared to the past.

### 3.4 Theme 4: Hope for future intragroup progress within the current system

In this theme, the findings reveal that participants believe female referees and observers currently have better rights and opportunities and are more represented than before. Additionally, participants expressed optimism that conditions for female referees and observers will improve even further in the future. They emphasized the increasing presence of women in the leadership roles of the Turkish Football Federation. For instance, X shared their thoughts on this issue:

“I think women now have more of a voice. For instance, we even have a female member on the Central Referee Committee. This wasn’t the case in the past. It shows that women are being valued and that they also have a say. I hope this continues.”

These findings indicate that participants have aligned with a system-justifying tendency. According to the theory of system justification, disadvantaged individuals or groups may internalize their conditions and perceive the existing structure as legitimate and fair through prevailing ideologies and stereotypes (Jost and ve Hunyady, 2005). Although participants previously acknowledged being marginalized due to their gender, in this theme, they perceive no institutional discrimination and believe future conditions will be more advantageous for women. This reflects their tendency to justify the system.

Many referees have other full-time careers and often referee “for the love of the sport.” Therefore, the belief that the structure will continue to improve may help referees remain committed to their profession and cope with the occasional challenges and barriers they face.

After identifying the main emerging themes, the researchers re-examined all transcripts to identify other transcripts that fit the new themes. As a result of this process, four themes emerged that were present in 100% of the interviews. A full list of stated and illustrated themes and first-level codes is shown in Table 2.

**Table 2:** Examples of codes and themes used during thematic analysis of the study.

TEMA	CODE	EXCERPT
The hope that future intra-group advancement will be possible within the current system.	Positive change Female member in MHK and Federation support is equal and fair There are no institutional barriers, there is development, there will be further development, rapid acceleration This was not the case in the past (more obstacles and pressure in the past), Equal opportunities now compared to the past TFF to publish equal instructions for men and women without discrimination,	Nilay: “I mean, I think women have more say now. For example, we even have a woman member in the Central Referee Board, which was not the case in the past, this shows that women are valued and they have a say. Therefore, I want this practice to continue.” Oya: “If women’s football develops, women’s refereeing develops and at some point women’s football also develops, so the federation is doing its part and supporting women. It puts women managers in every organization, so I think the situation is much better now than before.”
Legitimating Myths That Reduce Hierarchy	A more moderate point of view of fans, managers, coaches and athletes and a little bit of fan pressure Societal perspective: more rigid in the past, more constructive now, better than before There are positive developments. Women have been recognized in this field. No prejudice like before. More polite welcome and acceptance by field managers, field commissioners, facility supervisors, football representatives than men	Zeynep: … “*but in the past it was different, now those taboos have been broken, so the trend is good”: Breaking down sexist taboos over time In the past, of course, there were more spatial problems, but now we see that they have been overcome, now that there is a women referee dressing room, I think this is a great start.* Simge: It is in a much better position than it used to be, so people do not have as much prejudice as before, so now women can play ball, women can comment on football, women can referee.
Glass Ceiling: Organizational Factors	Not wanting female referees and observers in men’s matches (club pressure, obstacles) Not getting match assignments in top leagues; Stepchild treatment, discrimination, being put in the second plan (subordination, marginalization), Taking a long time to climb the career ladder	Ecem: “no coach used to want a woman referee*”* Sude: “There is gender-based injustice, I was always offered but not taken up”
Gender-based positive and negative experiences	Gender advantages: more positive approach, no swearing Courageous refereeing Celebrating bravery (players); Bias	Oya: “we female football observers trivialize or ignore sexist abuse by accepting (legitimizing) gender stereotypes as a fundamental part of the game.

## 4 Conclusions and recommendations

This study examines how female football observers in Turkey adapt to societal pressures and the glass ceiling barriers through tendencies to legitimize the system, despite experiencing these challenges. Football has historically been a male-dominated field, creating an environment that shapes institutions, including sports refereeing, and reinforces traditional power structures that limit women’s participation (Fields, 2005). Being present in this male-dominated domain is challenging for women, as evidenced by Nordstrom et al. (2016) work, which highlights the difficulties faced by female football referees. Similarly, this study reveals that female observers encounter comparable experiences, framed within the theories of system justification, social pressure, and the glass ceiling.

The results of this study highlight four main themes that emerged from the participants’ experiences in line with the aforementioned theories: gender-based positive and negative experiences; the glass ceiling and organizational factors; hierarchy-reducing legitimizing myths; and hope for future intragroup progress within the current system. The findings demonstrate that women’s experiences as football observers are strongly aligned with the general norms of system justification, particularly in relation to gender-based system legitimation. The research shows that sexist discourse and behaviors, as well as institutional policies and practices, are legitimized within this context.

Theberge (1993) in her research on female sports coaches, notes that many women working in sports are aware of their minority status and face problems, such as negative stereotypes and biases from male athletes, coaches, and referees. This study also finds that what is legitimized here is the entrenched and accepted status of female referees and observers within the male-dominated football culture. Two key themes emerged as part of this legitimizing action: *gender-based positive and negative experiences* and *hope for future intragroup progress within the current system*. Hacicaferoğlu and Ve Gündoğdu (2014) research on referees shows that female football referees are subjected to more suppressive behaviors compared to their male counterparts.

Moreover, other studies indicate that women often downplay sexist remarks, describing them as jokes or light-hearted, even when they recognize the sexist nature of the comments. This trivialization allows sexist interactions to persist without challenge, reinforcing male dominance (Forbes et al., 2015). Webb et al. (2020) suggest in their research that some referee participants believe football has developed a culture where such actions by players, coaches, and managers are understood as a normal part of football culture.

The findings of this study also show that participants experienced social pressure, particularly from fans and coaches, during the early stages of their refereeing and observing careers. Any negative decision against a team would lead fans to turn immediately to the observer in the stands, directing negative remarks at them. This was especially true for female referees, as many believed women should not officiate men’s matches. Participants expressed that they had to fight for acceptance in this male-dominated space. This aligns with Cleland et al. (2018) research, which found that female referees, operating in a domain dominated by men and masculinity, often had to continually negotiate their identity as women in the face of mistreatment from managers, players, coaches, and spectators. However, as their refereeing skills improved, they began to receive more recognition for their abilities from players, coaches, and spectators. Similarly, female observers noted that they now receive more positive support from their colleagues and institutions than in the past.

Kellett and Shilbury (2007) and Kellett and Warner (2011) emphasize that the strength of social experiences and relationships within institutions is crucial for referees’ career sustainability and ongoing participation, contributing to enduring connections and commitment. Given the male-dominated nature of refereeing (Todey, 2011), participants mentioned that, despite social pressures from spectators and coaches, the support they received from colleagues and institutions increased their motivation for their work.

In terms of glass ceiling barriers, the study finds that the obstacles female referees face are primarily rooted in organizational factors. One of the key findings is that it is much more difficult for female referees to officiate in higher leagues compared to their male counterparts, highlighting the components of the glass ceiling that prevent women from advancing to higher ranks. Participants noted that they progress through their careers much more slowly than their male colleagues and that women’s presence is often overlooked when it comes to top-league assignments, where decisions are frequently based on gender rather than ability.

Another significant finding of the research is that participants believe that being a female referee or observer was much more challenging in the past. However, they also recognize that important progress has been made in women’s football in Turkey, leading to better representation in their professions. In other words, participants indicated that they are now in a better position, with increased representation, greater societal and institutional acceptance, but that organizational factors still perpetuate glass ceiling barriers.

The authors suggest that by identifying elements involved in legitimizing actions, a better understanding of sports policy processes may emerge. Therefore, the authors encourage the identification of legitimizing actions and associated elements across various contexts. Consistent examination and implementation of positive policies for female football referees can increase their level of empowerment, reduce the gender gap, and create a productive force in challenging traditional gender inequalities by reducing or even eliminating tendencies to legitimize them.

The findings of this study help us understand the challenges faced by female observers and propose policies that could minimize the loss of human resources in the field of sports refereeing and observing. Consistent examination and implementation of positive policies for female football referees can increase their empowerment, reduce gender disparities, and create a productive force in addressing traditional gender inequalities (Kim and Hong, 2016). To ensure equal advancement within the system for female football referees and observers in Turkey and to prevent glass ceiling barriers, it is recommended that the Turkish Football Federation develop institutional policies aimed at gender equality. Furthermore, developing strategies to attract more female athletes, especially those participating in sports at high schools and universities, to the refereeing role would be beneficial.

## Data Availability

The data analyzed in this study is subject to the following licenses/restrictions: Since this research is a qualitative study, the interviews were recorded and later reported. Therefore, the data of the participants are limited because they are their personal opinions. Requests to access these datasets should be directed to Yeliz Eratlı Şirin, yelizsirin75@gmail.com.
